# Shear Thickening, Star-Shaped Polymer Electrolytes for Lithium-Ion Batteries

**DOI:** 10.3390/molecules29163782

**Published:** 2024-08-09

**Authors:** Magdalena Słojewska, Arkadiusz Czerwiński, Marcin Kaczorowski, Ewa Zygadło-Monikowska

**Affiliations:** Faculty of Chemistry, Warsaw University of Technology, Noakowskiego 3, 00-664 Warsaw, Poland; magdalena.slojewska.dokt@pw.edu.pl (M.S.); arkadiusz.czerwinski.dokt@pw.edu.pl (A.C.); marcin.kaczorowski@ichp.lukasiewicz.gov.pl (M.K.)

**Keywords:** li-ion, safe lithium-ion battery, shear thickening electrolyte, polymer electrolytes, impact resistant batteries

## Abstract

The safety concerns associated with current lithium-ion batteries are a significant drawback. A short-circuit within the battery’s internal components, such as those caused by a car accident, can lead to ignition or even explosion. To address this issue, a polymer shear thickening electrolyte, free from flammable solvents, has been developed. It comprises a star-shaped oligomer derived from a trimethylolpropane (TMP) core and polyether chains, along with the inclusion of 20 wt.% nanosilica. Notably, the star-shaped oligomer serves a dual function as both the solvent for the lithium salt and the continuous phase of the shear thickening fluid. The obtained electrolytes exhibit an ionic conductivity of the order of 10^−6^ S cm^−1^ at 20 °C and 10^−4^ S cm^−1^ at 80 °C, with a high Li^+^ transference number (t_+_ = 0.79). A nearly thirtyfold increase in viscosity to a value of 1187 Pa s at 25 °C and a critical shear rate of 2 s^−1^ were achieved. During impact, this electrolyte could enhance cell safety by preventing electrode short-circuiting.

## 1. Introduction

Lithium-ion batteries (LIBs) currently dominate the market as power sources for mobile electronic devices, such as laptops, mobile phones, and so forth. In addition, LIBs have been the dominant power sources for electric vehicles (EVs) in recent years [1]. EVs powered by electrical energy require the installation of several thousand cells in each device. Despite advanced solutions in cell temperature management, battery fires and explosions have still been observed in recent electric vehicle accidents [2]. In these accidents, impact-induced penetration of the liquid electrolyte and porous polymer separator caused an internal short circuit of the electrodes, generating a large amount of heat, which ultimately resulted in fire and explosion. The greatest challenges related to the lithium-ion cells’ upgrades are improvements in their safety by eliminating organic solvents present in classic, non-aqueous electrolytes. For this reason, currently produced LIBs are packed, e.g., in steel cans, which protect the cell against mechanical damage and can also withstand high internal pressure [2,3,4,5,6]. However, the addition of heavy packaging significantly lowers the gravimetric and volumetric energy density of the cell. Rather than introducing a heavy external component, a solution focused on optimizing the composition of the battery itself would be preferable. One of the solutions is to replace the flammable, volatile carbonates with other solvents, but although many different alternative liquid electrolytes have been proposed so far, they still require a lot of research [7,8]. As a result, carbonates are still in use in most of the cells produced nowadays. In order to improve the specific properties of the electrolyte or reduce many of their problems, flame retardants and other additives are used [9,10,11]. Unfortunately, this is only a partial solution and does not make it completely safe to use or does not have the appropriate electrochemical properties to be used in production. Another method is to replace the liquid systems with solid polymer electrolytes (SPEs) or immobilize the solvent molecules in gel polymer electrolytes (GPEs) [12,13]. Although SPEs fully comply with safety requirements, their use is still limited due to their low ionic conductivity, poor compatibility with electrodes, or other limitations [14]. 

A different approach to the safety of electrolytes would be the development of systems capable of responding to an impact. Such systems are currently being investigated and their operation is based on the shear thickening effect. Shear thickening fluids (STFs) (also called dilatant fluids) are colloidal suspensions that exhibit a significant increase in viscosity with increasing shear rate [15]. This process is reversible, so the material regains its original properties after the force is removed. Due to such properties and their ability to absorb energy, STFs are good candidates for protective applications, for example, in liquid armor and protective clothing [16,17,18], shock absorbers and dampers [19,20,21,22], and protective sports equipment [23,24]. STFs usually consist of colloidal particles of a size up to 100 microns in diameter (e.g., silica, calcium carbonate, and so on) suspended in a carrier liquid (e.g., water, ethylene glycol, and polyethylene glycol). The introduction of the lithium salt into STFs forms a new type of electrolytes called shear thickening electrolytes (STEs). These electrolytes could exhibit high conductivity values similar to those of liquid electrolytes while simultaneously possessing better mechanical properties, particularly under impact, akin to solid electrolytes [25]. It is this transition to a solid-like viscosity that prevents the electrodes from short-circuiting during an impact, such as in a car accident, dramatically improving the safety of LIBs.

The first report about the use of shear thickening electrolytes for lithium-ion battery application belongs to Ding et al., who described systems containing fumed silica and 1 M LiFP_6_ in EC/DMC. The electrolyte with 9.1 wt.% silica showed shear thickening behavior, with an increased ionic conductivity compared with the commercial electrolyte [26]. Veith et al. also studied silica nanoparticles introduced into conventional liquid electrolytes, introducing the concept of SAFIRE–Safe Impact Resistant Electrolytes, which can be produced from battery-compatible and low-cost materials. Studies of various types of synthesized and commercially purchased silicas show that the internal short circuit may be prevented by the application of STFs [27]. However, so far, all these shear thickening electrolytes have contained low-molecular-weight, flammable carbonates that have low boiling points and high vapor pressures. This is not the most advantageous option due to the fact that during the shear thickening effect, kinetic energy is converted into other types of energy, such as thermal energy [28]. This may cause the cell to burst due to heating. 

In electrolytes based on organic carbonates, it has been observed that silica colloids in suspension aggregate over time and even precipitate from the solution. Veith et al. proposed reducing sedimentation and improving dispersion stability by using surface-tethered polymeric brushes synthesized via surface-initiated atom transfer radical polymerization [29]. The phenomenon of flocculation is not observed in composite systems where polymer is used as the continuous phase. This is undoubtedly an advantage, considering the complicated and time-consuming process of surface functionalization of colloidal silica particles [30].

From the perspective of ionic conductivity in lithium polymer electrolytes, among the polymers studied so far, polyethers exhibit the optimal properties and are the most frequently applied polymer matrix [31]. The results of studies on the dependence of polymer viscosity on chain structure indicate that polyethers with a branched, star-shaped architecture exhibit lower viscosity compared to their linear analogues of the same molar mass. Star-shaped branching significantly affects the shear sensitivity of the bulk viscosity [32]. For this reason, star-shaped polyether polymers should exhibit better conductivity properties because the mobility of ions and the associated ionic conductivity significantly depend on the viscosity of the medium in which the ions move. This structure of continuous phase molecules introduces another advantageous factor. The concentration of hydroxyl groups, which are the end groups of polymers, is higher in the case of branched structures. This should have a beneficial effect because hydroxyl groups are involved in the formation of hydrogen bonds, which are crucial for the shear thickening effect.

In the present study, we aim to present the properties of new composite electrolyte systems doped with commercially available nanosilica and synthesized star-shaped polyethers exhibiting the properties of shear thickening fluids. We discuss their potential use in lithium-ion batteries with enhanced safety.

## 2. Results and Discussion

### 2.1. Star-Shaped Oxyethylene and Oxypropylene Glycols

**Figure 1:**
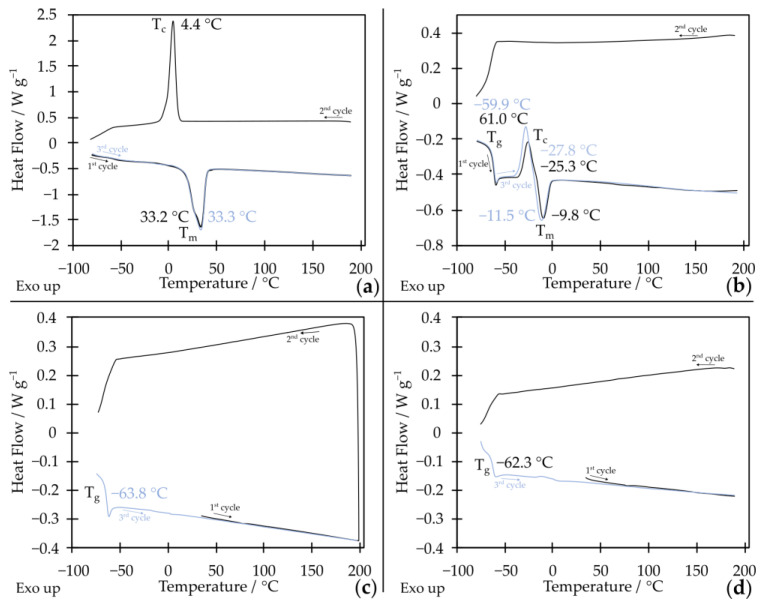
DSC thermograms of star-shaped (**a**) oxyethylene glycol with a molar mass of approximately 2000 g mol^−1^; (**b**) oxyethylene glycol with a molar mass of approximately 1000 g mol^−1^; (**c**) oxypropylene glycol with a molar mass of approximately 2000 g mol^−1^; (**d**) shear thickening fluid made of star-shaped oligo(oxypropylene) (STF1-star-PPG).

The product, with a mass of approximately 2000 g mol^−1^, obtained according to the synthesis described in Figure 1, where the monomer was ethylene oxide, was a solid at room temperature. In the thermogram, we observe the melting of the crystalline phase at a peak temperature of T_m_ = 33.3 °C (Figure 1a). In the cooling cycle, we can observe the crystallization of this oligomer at a temperature of T_c_ = 4.4 °C (Figure 1a). Therefore, it was decided to obtain star-shaped oligo(oxyethylene) with a lower molar mass. Star-shaped oligo(oxyethylene) with a mass of approximately 1000 g mol^−1^ at room temperature has the properties of a viscous liquid. However, it is not fully amorphous. Cold crystallization, which in this case we observe during heating cycles, occurs at a temperature of −27.8 °C. This phase then melts at T_m_ = −11.5 °C (values determined from the second heating cycle, Figure 1b), which means it cannot be used in a very wide temperature range. However, the product, whose repeating units are propylene oxide, has the properties of a viscous liquid at room temperature. It is fully amorphous and characterized by a glass transition temperature of −63.8 °C (Figure 1c). Thanks to this, it can be used in a wide temperature range. This is important in the case of using star-shaped oligo(propylene glycol) as a solvent in shear thickening electrolytes, which are to be used in li-ion cells. 

^1^H, ^13^C NMR, and MALDI-TOF spectroscopy methods confirmed the obtainment of the expected product as a result of anionic polymerization on a core derived from potassium alkoxide obtained by reaction with TMP. The spectrum shows signals from protons in the −CH_3_ (0.74–0.85 ppm), −CH_2_− (1.28–1.40 ppm), and −CH_2_O− (3.21–3.30 ppm) groups from the initiator constituting the core of the oligomer, as well as signals from the groups −OCH_2_ (3.55–3.63 ppm) or −OCH− (3.47 ppm) coming from the oligomer arms. Signals from protons in the last repeating unit are also visible at 3.60–3.95 ppm. In the case of three-armed propylene glycol, a signal from the −CH_3_ group in the chain (1.16 ppm) is also visible (Figure S1). Moreover, from the ^13^C NMR spectra, it can be concluded that each hydroxyl group of the TMP core is substituted based on the 43 ppm unsplit signal (Figure S2). MALDI-Tof analysis (Figure S3) allowed us to confirm that the tested product contains mainly the 3-arm oligo(oxypropylene).

### 2.2. Shear Thickening Fluids

**Figure 2:**
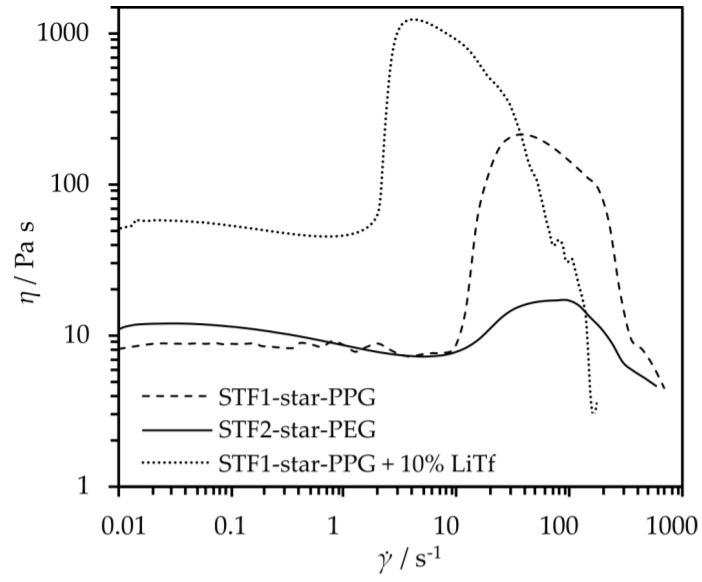
Viscosity curves of fluids made of star-shaped oligo(oxypropylene) (STF1-star-PPG), oli-go(oxyethylene) (STF2-star-PEG), and the electrolyte containing 10% LiTf (lithium triflate) made from STF1-star-PPG.

In order to determine the influence of the continuous phase on the rheological properties of fluids, the obtained star-shaped glycol-based composites were subjected to rheo-logical tests. The test results are presented in Figure 2. Both types of tested composites, the one obtained with star-shaped oligo(oxypropylene) (STF1-star-PPG) and the one with oligo(oxyethylene) (STF2-star-PEG), are shear thickening fluids. The viscosity jump is observed at a similar value to the critical shear rate. However, the value of the viscosity jump is much higher for a fluid made of STF1-star-PPG (205 Pa s) than for STF2-star-PEG (10 Pa s). 

**Figure 3:**
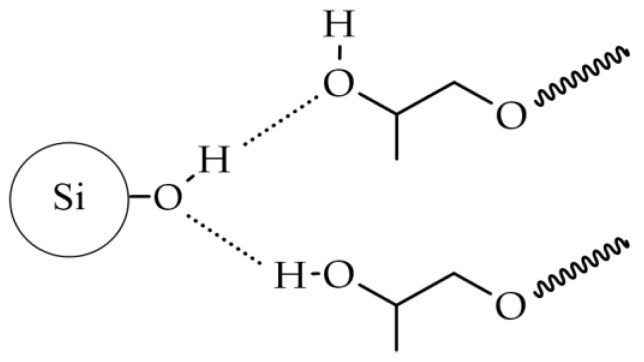
Intermolecular hydrogen bonding between hydroxyl groups of propylene glycols and silanol groups on the surface of nanosilica.

To interpret the observed effect for the studied dispersion system, one can refer to the theory describing the phenomenon of shear thickening. Currently, several theories exist, all of which assume a change in the internal structure of the shear thickening fluid after exceeding a critical shear rate, leading to an increase in flow resistance and, consequently, an increase in the system’s viscosity. The widely accepted and most commonly used theory to describe phenomena occurring in shear thickening systems is the theory of hydroclusters [33]. It states that after surpassing the critical shear rate, hydrodynamic attractive forces begin to dominate over repulsive Brownian forces. This leads to the agglomeration of solid phase particles and the formation of so-called hydroclusters. These large agglomerates disrupt the orderly structure of the fluid and collide with each other, resulting in an increase in viscosity. For dispersions containing silica, it is generally assumed that in strongly hydrogen-bonding liquids, a solvation layer forms on the silica surface through hydrogen bonding between the liquid molecules and surface silanol groups (Si−OH) [34]. The change in viscosity is attributed to the interaction of the hydroxyl groups present at the ends of the chain with the nanosilica surface, as shown in Figure 3.

Additionally, the viscosity of the system at low shear rates is lower for star-shaped oligo(oxypropylene). This is a desirable feature in the context of using composites as electrolytes for li-ion cells because the ionic conductivity is closely related to the viscosity of the system. Due to the change in viscosity over twenty times greater for a fluid made of 20 wt.% SiO_2_ with star-shaped oligo(oxypropylene), we decided to obtain electrolytes from it and further analyze them. Moreover, the shear thickening fluid made of star-shaped oligo(oxypropylene), like the oligomer itself, does not crystallize in the entire range of tested temperatures. It is characterized by a low glass transition temperature of −62.3 °C (Figure 1d). Therefore, nanosilica does not act as crystallization nuclei, and the composite can retain its properties typical of a fluid over a wide temperature range.

### 2.3. Electrolytes Based on Star-Shaped Glycols

**Figure 4:**
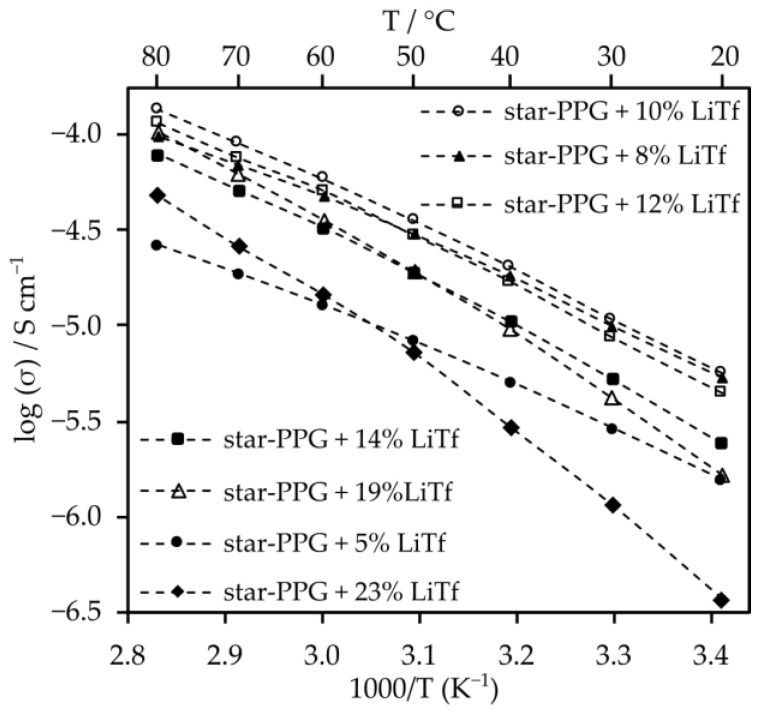
Ionic conductivity of electrolytes made of star-shaped oligo(oxypropylene) (star-PPG) and lithium salt.

Measurements were carried out to determine the optimal salt content for electrolytes consisting only of star-shaped oxypropylene glycol. The analysis indicates that the optimal salt content in this solvent, which provided the highest conductivity values across the entire range of tested temperatures, is 10 wt%. Additionally, the conductivity of 10% LiTf solution at 20 °C is 5.1 × 10^−6^ S cm^−1^. However, at 80 °C, it increases to 1.2 × 10^−4^ S cm^−1^ (Figure 4).

**Figure 5:**
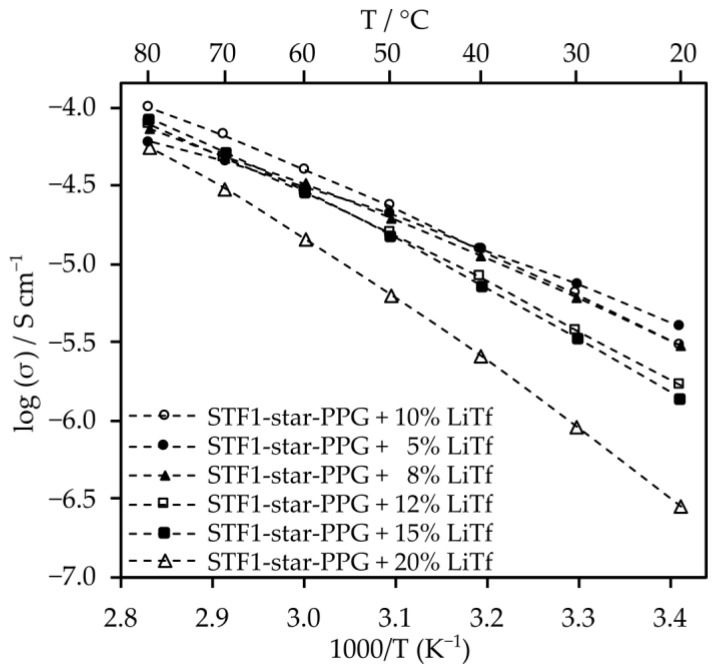
Ionic conductivity of electrolytes made from STF1-star-PPG and lithium salt.

The optimization of salt content for electrolytes that contain the addition of nanosilica was also conducted (Figure 5).

**Figure 6:**
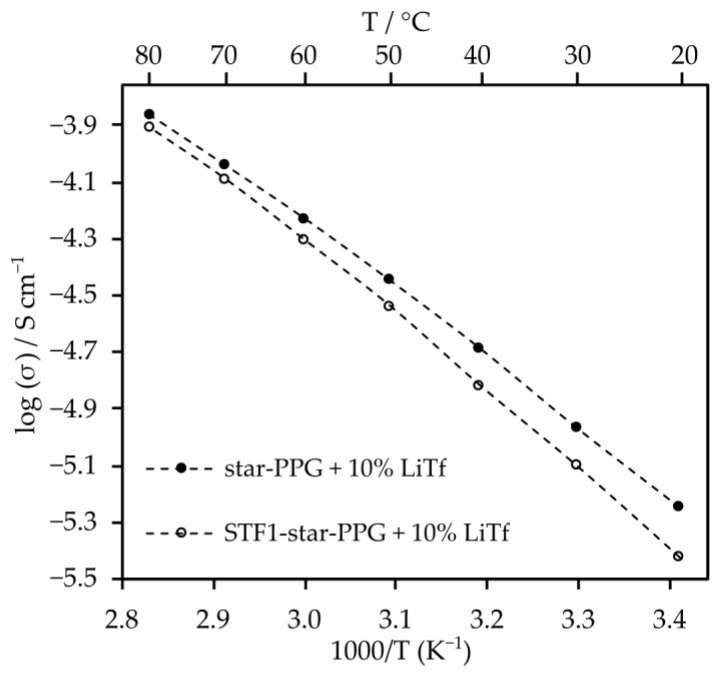
The effect of adding silica to star-PPG on ionic conductivity.

Electrolytes with 10% salt content by weight exhibit similar conductivity values to those with 8% and 5% salt content. Moreover, at temperatures exceeding 40 °C, their conductivity surpasses that of electrolytes with other salt contents. Despite the significant viscosity of the composite electrolyte, the inclusion of nanosilica induces minor changes in conductivity (Figure 6). This is likely attributed to enhanced lithium ion mobility resulting from Lewis acid–base interactions between SiO_2_ particles and salt anions. Furthermore, such electrolytes exhibit very high transference numbers, which are 0.79 for the system with silica and 0.85 for the electrolyte without silica. 

We attribute this high transference number (t_+_ = 0.85) to the structure of the oligomer itself, which contains hydroxyl groups at the ends of the oligoether arms. These groups can form hydrogen bonds between the star oligomer and the anions of the lithium salt. The anions of the triflate salt we used (CF_3_SO_3_^(−)^) contain several highly electronegative C−F groups, which can form extensive hydrogen-bond connections with the hydroxyl groups in the oligomer [35]. The hydrogen bonds occurring in the system between the salt anions and the electrolyte matrix are responsible for the significant immobilization of the anions. This effect related to the immobilization of anions due to the formation of hydrogen bonds has been noticed and described for poly(vinylidene fluoride-hexafluoropropylene)-based solid-state electrolytes.

Our research shows that the introduction of silica with silanol groups on the surface of the particles slightly weakens this effect, as some of the end groups of the oligomer interact with the silica surface. The transference number remains high but is lower than in the system without silica (t_+_ = 0.79).

**Figure 7:**
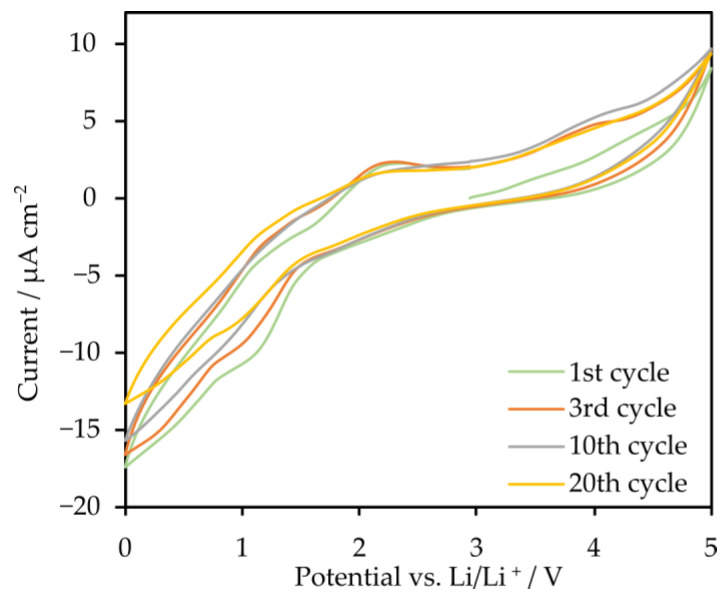
Cyclic voltammetry (CV) curves of STF1-star-PPG + 10% LiTf.

Cyclic voltammetry (CV) in a three-electrode system was used to investigate the reactivity of the electrolyte within the voltage range of 0–5 V vs. Li/Li^+^ (Figure 7) at a scanning rate of 1 mV/s. Cathodic peaks were observed in the ranges of 0.6–1.1 V and 1.2–1.8 V. These peaks are likely associated with the reversible incorporation of lithium into the electrode and the formation of the solid electrolyte interphase (SEI) layer on its surface. In the voltage range above 3.7 V up to approximately 4.4 V, a peak appears, which can be attributed to irreversible electrolyte decomposition. The overlap of the curves in subsequent cycles indicates that the electrode exhibits stable cycling performance. CV measurements indicate that the electrolyte is electrochemically stable up to a potential value of 3.7 V relative to lithium. Above this value, the electrolyte begins to decompose.

**Figure 8:**
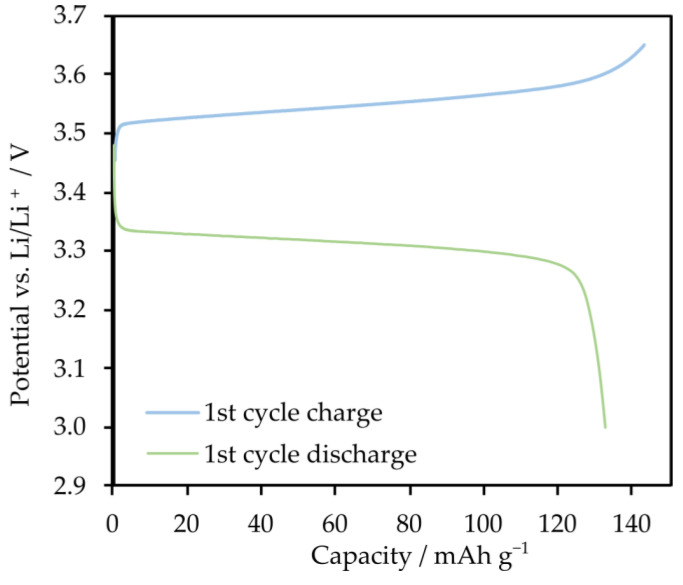
The first cycle charge/discharge characteristics of the Li|STF1-star-PPG|LFP half-cell.

Based on the cyclic voltammetry results, we explored the potential application of the proposed electrolyte in a lithium cell with a LiFePO_4_ (LFP) cathode. This choice was driven by the lower charging voltage of the LFP cathode compared to the potential decomposition voltage of the electrolyte. Figure 8 illustrates the first cycle of charging and discharging in a Swagelok-type cell. The cell was charged to a voltage of 3.65 V and discharged to 3.0 V, both at a constant current of C/30. The cell achieved a capacity of 133 mAh/g with a Coulombic efficiency of 93%. This high efficiency indicates that the proposed electrolyte may be compatible with the LFP cathode, providing stable and efficient performance within the operational voltage range.

**Figure 9:**
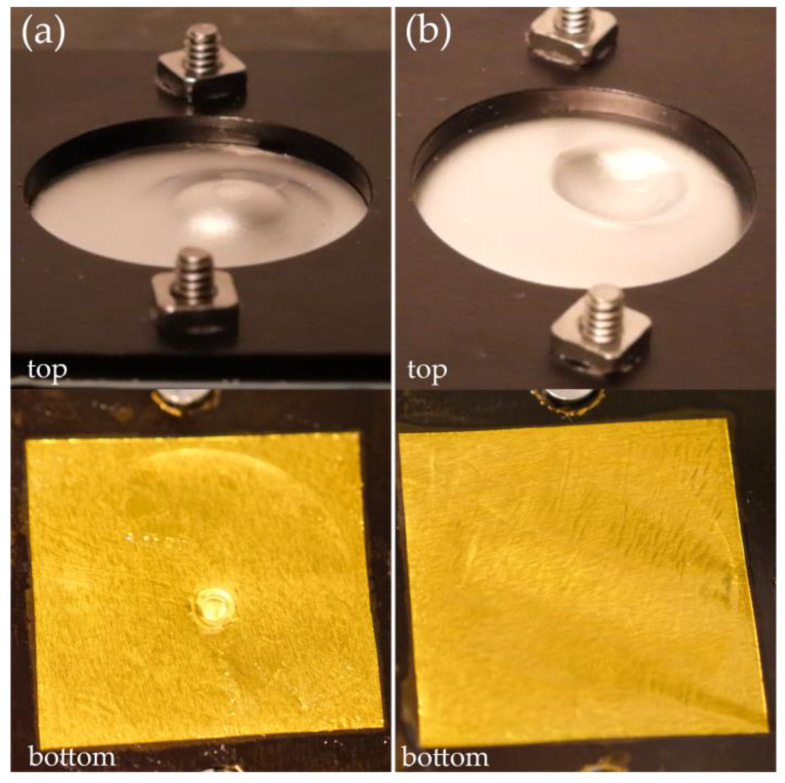
Foils after being hit with the steel ball: (**a**) the electrolyte containing EC/DMC; (**b**) the shear thickening electrolyte; “top” refers to the upper layer that was directly hit by the ball; “bottom” refers to the lower layer, separated by the electrolyte.

Furthermore, the rheological tests depicted in Figure 2 demonstrate that electrolytes containing, in addition to lithium triflate, nanosilica and star-shaped propylene glycol exhibit a viscosity jump of 1187 Pa s, which is a value approximately 19 times higher than that of an electrolyte containing a mixture of ethylene carbonate (EC) and dimethyl carbonate (DMC) as solvents [26]. Moreover, a steel ball in free fall was unable to penetrate the shear thickening electrolyte we made. Therefore, unlike the electrolyte containing classic low-molecular-weight solvents, the shear thickening electrolyte protected the system against the short-circuiting of both foils. This is illustrated in Figure 9, which shows photos of the foil taken after the experiment. Clear traces are visible (Figure 9a), proving that the falling ball was able to short-circuit the foils in the electrolyte containing EC/DMC. In the case of the shear thickening electrolyte (Figure 9b), after the ball fell, it left only a small indentation in the top foil, while the foil at the bottom remained undamaged. 

**Figure 10:**
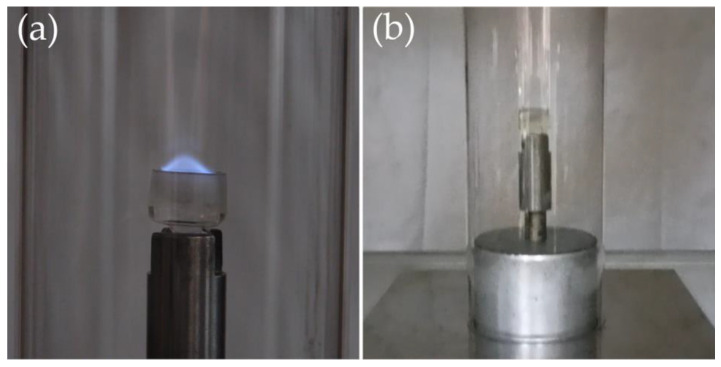
Flammability of materials: (**a**) the electrolyte containing EC/DMC; (**b**) the shear thickening electrolyte containing 3-armed star-shaped oxypropylene glycol.

What is more, tests have shown that the shear thickening electrolyte, unlike the electrolyte containing an EC/DMC solvent mixture, is non-flammable at an oxygen content corresponding to its concentration in air (Figure 10). The electrolyte containing EC/DMC first burns with a very bright blue flame, and only after some time, when a certain amount of the solvent has burned out, does the flame turn red, characteristic of lithium. In contrast, the shear thickening electrolyte starts burning only when the oxygen content in the gas mixture reaches 40%. This behavior is due to the use of star-shaped oxypropylene glycol as a solvent, which has a negligibly low vapor pressure. Additionally, the Oxygen Index Value for the most commonly used polymer in electrolytes, poly(ethylene oxide), is 15% [36]. The structure of the repeating unit (an additional −CH_3_ group in the chain) and, above all, the addition of silica, which acts as a flame retardant, increase the oxygen concentration at which the material ignites. 

## 3. Materials and Methods

### 3.1. Chemical and Reagents

Propylene oxide (99%), ethylene oxide (99.8%), potassium (99.5%), 1,1,1-tris(hydroxymethyl)propane (98%), tetrahydrofuran (anhydrous, 99.9%) silica (declared grain size 7 nm), AmberLite IR120 ion exchange resin, ethylene carbonate (anhydrous, 99%), and dimethyl carbonate (anhydrous, 99%) were purchased from Sigma-Aldrich (St. Louis, MO, USA) and used without further purification. Methanol (analytical grade) was purchased from POCH. Lithium trifluoromethanesulfonate (LiTf, LiCF_3_SO_3_) (96%) was purchased from Sigma-Aldrich, dried under reduced pressure at 130°C, and kept in an argon atmosphere. Metallic lithium was used in the form of a ribbon, 1.5 mm thick and 100 mm wide, purchased from Sigma-Aldrich. LiFePO_4_ cathode powder was purchased from TOB New Energy, Poly(vinylidene fluoride) powder was purchased from Alfa Aesar, Vulcan XC72R Carbon Black was purchased from Cabot, and N-Methyl-2-pyrrolidone (NMP, 99.8%) was purchased from Roth.

### 3.2. Synthesis of Star-Shaped Oxyethylene and Oxypropylene Glycols

**Figure 11:**
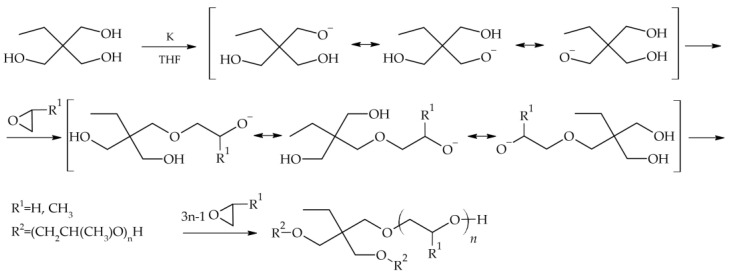
Reaction scheme for synthesizing star-shaped oligomers.

Star-shaped glycols have been obtained using an anionic polymerization mechanism (Figure 11). Trimethylolpropane (TMP) (2.84 g) was used as the core. The reaction was carried out in a pressurized reactor under an inert gas atmosphere. The synthesis proceeded in two stages. The first stage was initiation. Metallic potassium, which was in deficiency (0.25 g, 10 mol% in relation to the TMP hydroxyl groups), was used as the initiator. Active alkoxide centers remained in equilibrium, which made it possible to obtain oligomeric stars with arms of equal length. In the case of ethylene glycols, this step was carried out in THF (10 mL) at room temperature for several hours. The mixture was then cooled using dry ice, and ethylene oxide was introduced. This reaction stage lasted for 10 h at a temperature of 50–60 °C. Star-shaped oligo(oxypropylene), on the other hand, was obtained without the presence of a solvent. The first stage of the reaction was carried out for 4 h at 100 °C. The reactor was then cooled to room temperature, and the appropriate amount of propylene oxide was introduced. Polymerization was carried out for 12 h at 140 °C. After the reaction was completed, the reaction mixture was purified by passing it through a column filled with a cation exchanger to remove potassium ions. Methanol was used as the eluent. After purification, the solvent was removed by vacuum distillation at 60 °C.

The amounts of reagents were selected to obtain an oligomer of propylene oxide (30.0 g) with a mass of approximately 2000 g mol^−1^ and ethylene oxide (39.5 g or 18.4 g) with masses of approximately 2000 g mol^−1^ and 1000 g mol^−1^, assuming complete conversion of the monomer.

### 3.3. Preparation of Electrolytes

Star-shaped oligo(oxypropylene) with a mass of approximately 2000 g mol^−1^ was used to obtain shear thickening electrolytes. Due to the fact that oligo(oxyethylene) with a similar mass is a solid at room temperature, it was decided to use a compound with a lower mass (approx. 1000 g mol^−1^). Nanosilica was added in portions to the obtained glycols while mixing the contents of the vessel using a mechanical mixer. When mixing was difficult due to the high viscosity of the system, the system was heated with a max temperature of 100 °C. In this way, shear thickening fluids were obtained. Lithium salt was added to the obtained STFs, and everything was mixed also using a mechanical mixer. The obtained composites were also placed in an ultrasonic bath. As a reference electrolyte, we obtained electrolytes without the addition of silica, and the appropriate glycol was mixed with salt. These activities were performed in an argon atmosphere.

The electrolyte containing low-molecular-weight organic solvents was also prepared in an argon atmosphere by mixing EC and DMC in a 1:1 volume ratio. Then, 10% by weight of lithium salts was dissolved in the carbonate mixture.

### 3.4. Experimental Techniques

Differential scanning calorimetry (DSC) was used to assess the type and quality of phase transformations occurring in the tested materials. Measurements were carried out on a Q2000 differential scanning calorimeter in the −80 to 200 °C temperature range with heating/cooling rate of 10 °C/min in hermetically closed aluminum vessels. All samples were prepared in an Ar-filled glovebox. 

The chemical structure of star-shaped glycols was characterized by ^1^H and ^13^C spectroscopy in solutions in CDCl_3_ (Varian Mercury VXR 400 MHz).

MALDI-Tof measurements were performed using an UltrafleXtreme mass spectrometer from Bruker Daltonics. Trans-2-[3-(4-tert-butylphenyl)-2-methyl-propenylidene]malononitrile (DCTB) was used as the matrix. Samples were dissolved in chloroform or THF.

Ionic conductivity of the electrolytes was determined via impedance measurements using a VSP-3e potentiostat (Bio-Logic, Seyssinet-Pariset, France) within the frequency range from 500 kHz to 1 Hz and the temperature range from 20 to 80 °C. The samples were stored in symmetrical cells with stainless steel electrodes. Ionic conductivity was calculated according to the following equation:

σ = l/(S × R),(1)

where R represents the resistance determined from the impedance measurement, S is the surface area of the electrolyte, and l is the thickness of the electrolyte. 

The transference number (t_+_) of lithium cations was determined using the polarization method with the Bruce–Vincent correction. According to this method, the electrolyte was placed in a symmetrical Li|electrolyte|Li system. A constant bias voltage of 20 mV was applied to the system. Then, the system was monitored until the polarization current reached the steady state (I_ss_ value). Additionally, impedance spectra were recorded before and after polarization. The following equation was used to calculate the transference number:

t_+_ = I_ss_ (U − I_0_R_0_)/[I_0_ (U − I_ss_R_ss_)], (2)

where U represents applied polarization potential, I_0_ and I_SS_ represent the initial and steady-state currents, respectively, and R_0_ and R_SS_ represent the corresponding initial and steady-state resistances of the solid-state interface calculated from the impedance plots before and after polarization.

Cyclic voltammetry (CV) was conducted in a three-electrode Swagelok-type system. The system included a stainless steel electrode as the working electrode (WE), a lithium electrode as the counter electrode (CE), and a platinum electrode as the reference electrode (RE). The measurements were carried out at a scanning speed of 1 mV/s within the voltage range of 0–5 V vs. Li/Li^+^. The measurements were performed at a temperature of 60 °C

The charge/discharge test was performed in a two-electrode swagelok-type cell. The cathode was a material composed of 80 wt.% LiFePO_4_ (LFP), 10 wt.% conductive carbon black, and 10 wt.% PVDF on an aluminum collector, while the anode was metallic lithium. The cathode was prepared using the slurry-casting method with N-methyl-2-pyrrolidone (NMP) as the solvent. After drying in a vacuum oven and pressing with a hydraulic press, the cathode had an average amount of cathode material (LFP) of 2.4 mg/cm^2^. The cell was thermostated at 60 °C and the open circuit potential (OCP) was measured. The first cycle started at the OCP value and continued until 3.65 V, after which the cell was discharged to a voltage of 3.00 V. Both charging and discharging were performed at a C/30 current rate, where 1C corresponds to 155 mA g^−1^ (the maximum capacity of LFP as declared by the manufacturer).

Steady-state rheological measurements were carried out using a Kinexus Pro rotational rheometer (Malvern Panalytical Ltd., Malvern, UK) operating in a plate–plate system with a plate with a diameter of 20 mm. The measurement temperature was 25 °C, and the width of the measurement gap was 0.3 mm. The measurement time was 5 min, measurement range 0.01–1000 s^−1^, and there were 10 measurement points per decade. Immediately before the measurement, the sample was sheared at an exponentially increasing shear rate from 0.01 to 0.1 s^−1^ for 2 min.

To determine the effectiveness of preventing our electrolyte from short-circuiting the electrodes, a drop test was conducted using a steel ball with a mass of 13.90 g and a diameter of 15 mm. The ball was dropped from a height of 0.5 m by releasing the electromagnet. The tested electrolyte was placed between two layers of aluminum foil (0.013 mm thick), which were mounted in a metal housing that protected the system from opening. The housing had a hole that allowed the ball to hit the sample.

The flammability of compounds was determined using the Oxygen Index apparatus (Fire Testing Technology, East Grinstead, UK). The sample, placed in a glass vessel, was ignited in a flow of gas (a mixture of oxygen and nitrogen). The first test was always conducted at an oxygen concentration similar to that found in air, i.e., 21%. Using a torch, attempts were made to ignite the sample for 5 s, repeated 6 times. If the sample was non-flammable, the oxygen content in the flowing gas was gradually increased and the operation was repeated. 

## 4. Conclusions

An innovative electrolyte for lithium-ion cells has been presented, designed to enhance the operational safety of lithium-ion batteries, particularly during impact. The developed electrolyte exhibits the properties of a shear thickening fluid and is free of flammable, low-molecular-weight organic solvents. The matrix used was synthesized via anionic polymerization, an approach that resulted in well-defined products that did not crystallize in the temperature range tested. Additionally, the synthesized three-armed oli-go(propylene oxide) is characterized by a low glass transition temperature of −64 °C. The shear thickening effect was achieved by incorporating a rheological modifier in the form of nanosilica. The lithium salt content (LiCF_3_SO_3_) was optimized to 10% by weight, yielding conductivities of 10^−6^ S cm^−1^ at 20 °C and 10^−4^ S cm^−1^ at 80 °C, along with exceptionally high transference numbers of 0.79, likely due to the oligomer’s structure. Furthermore, it was observed that the addition of silica had minimal impact on the electrochemical parameters of the electrolyte and the temperature of phase transitions. With a sufficiently high proportion of silica in the composite, it is possible to obtain an electrolyte with shear thickening fluid properties. The best system, based on star-shaped propylene glycol, exhibits a viscosity jump of nearly 1200 Pa·s, which significantly exceeds the values described in the literature so far. For STFs containing conventional electrolytes with nanosilica, the viscosity increase does not exceed 100 Pa·s [26].

Consequently, systems comprising branched oligo(propylene oxide) with silica and lithium salt represent a novel class of shear thickening electrolytes.

## 5. Patents

Zygadło-Monikowska, E.; Monikowska, D.; Kaczorowski, M.; Czerwiński, A.; Słojewska, M.; Burzyński, A. New polymer electrolytes with increased work safety and method of their preparation. PL243040B1, 27 June 2022. 

## Data Availability

Data can be available upon reasonable request.

## Supplementary Materials

The following supporting information can be downloaded at https://www.mdpi.com/article/10.3390/molecules29163782/s1, Figure S1: Spectra ^1^H NMR of star-shaped oxypropylene glycol obtained via anionic polymerization; Figure S2: Spectra ^13^C NMR of star-shaped oxypropylene glycol obtained via anionic polymerization; Figure S3: MALDI-ToF spectrogram of star-shaped oxypropylene glycol; Video S1: Drop test performed using a steel ball—the electrolyte containing EC/DMC; Video S2: Drop test performed using a steel ball—the shear thickening electrolyte; Video S3: Flammability of the electrolyte containing EC/DMC; Video S4: Flammability of the shear thickening electrolyte containing 3-armed star-shaped oxypropylene glycol–21% O_2_; Video S5: Flammability of the shear thickening electrolyte containing 3-armed star-shaped oxypropylene glycol–40% O_2_.
