# Supplementary material for: Shear Thickening, Star-Shaped Polymer Electrolytes for Lithium-Ion Batteries
Source: Molecules. 2024 Aug 9;29(16):3782. doi: 10.3390/molecules29163782 (PMC11357618; doi:10.3390/molecules29163782)
Supplement: Supplementary file 1 [file molecules-29-03782-s001.zip › Supplementary Materials.pdf]

Supplementary Materials

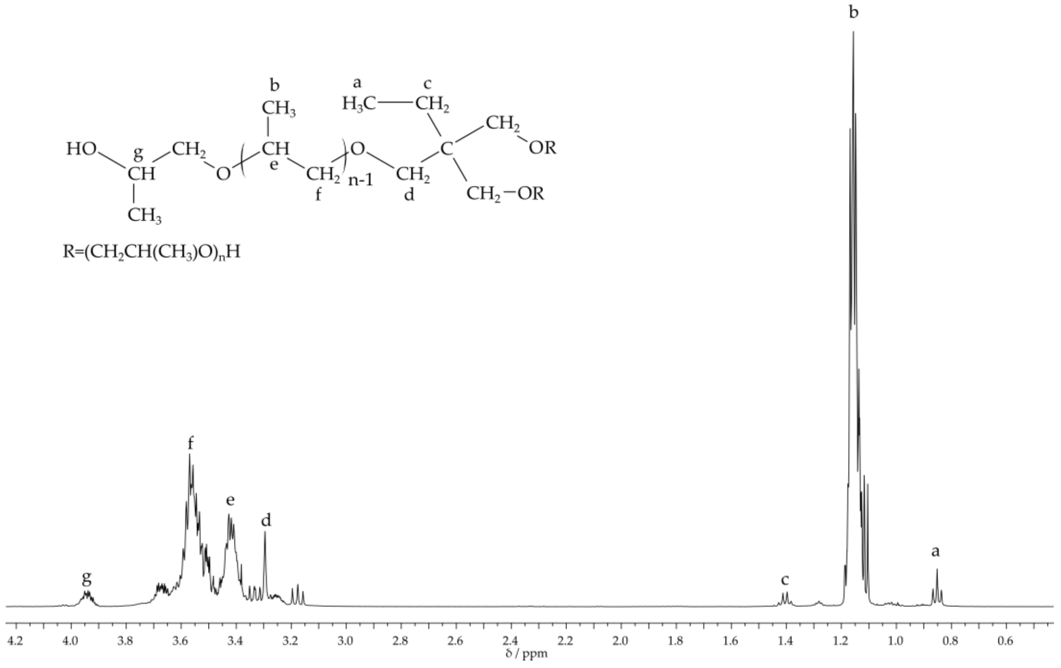

Figure S1. Spectra ^{1}H NMR of star-shaped oxypropylene glycol obtained via anionic polimerization.

^{1}H NMR ($CDCl_3$, 400 MHz): δ (ppm) = 0.84 (t, J=7.5 Hz, 3H, C**H**$_3$ in TMP), 1.08-1.18 (m, n × 3H, C**H**$_3$ in the chain), 1.38-1.40 (q, 2H, C**H**$_2$CH$_3$ in TMP), 3.31 (s, 6H, C**H**$_2$O in TMP), 3.31-3.47 (m, n × 1H, C**H** in the chain), 3.47-3.72 (m, n × 2H, C**H**$_2$ in the chain), 3.91-3.96 (m, C**H** from the end groups).

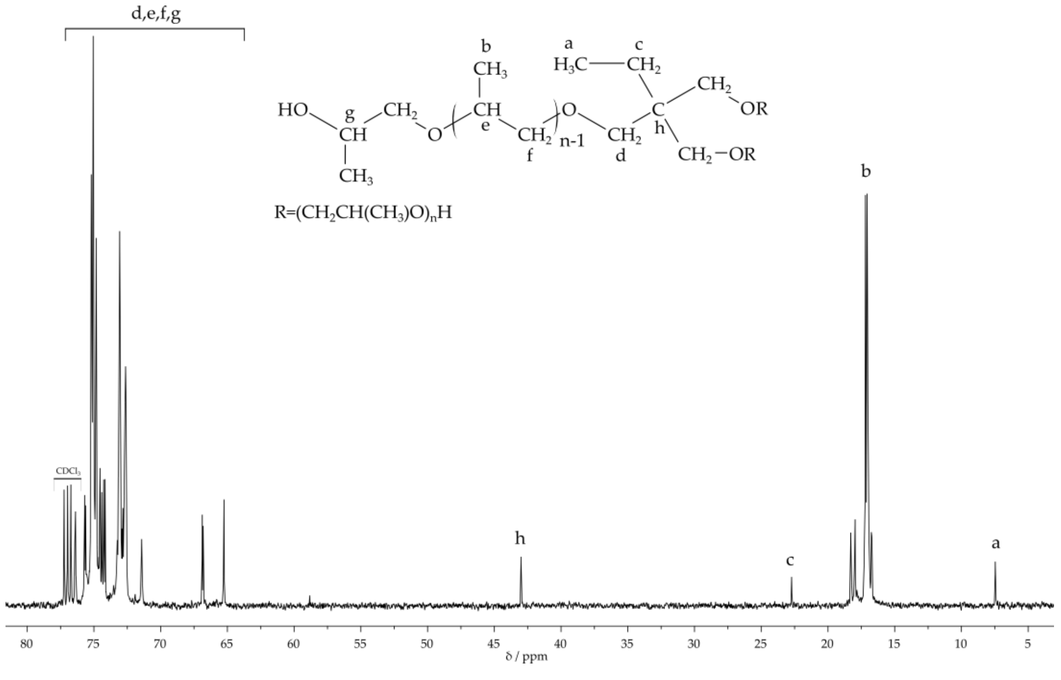

Figure S2. Spectra ^{13}C NMR of star-shaped oxypropylene glycol obtained via anionic polimerization.

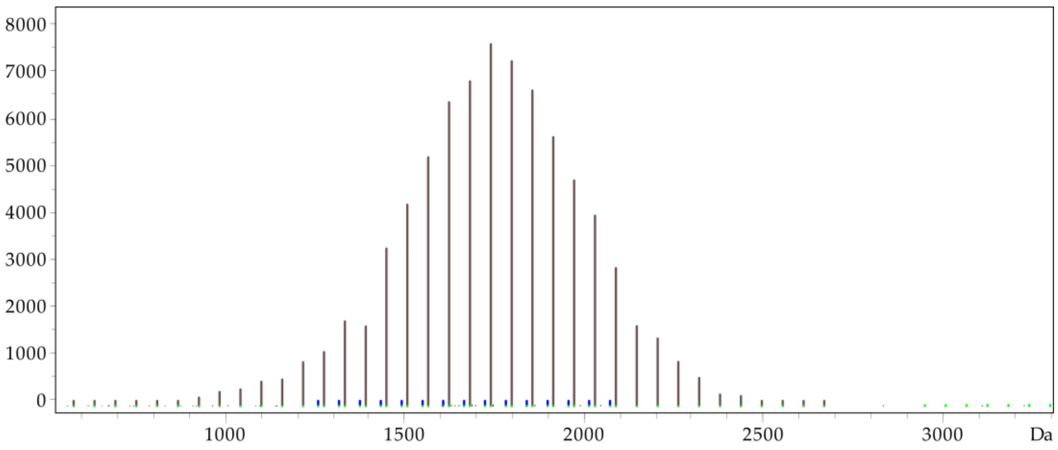

n	ser.	rep.unit	resid.	cation	Mn	Mw	pd	DP	% I.	cnt
1	1	58.0427	17.8897	38.9637	1705.17	1746.18	1.02405	29.3779	97.2	37
2	2	58.0427	1.91181	38.9637	1662.34	1686.86	1.01475	28.6399	0.6	15
3	3	58.0427	7.75796	38.9637	3334.50	3354.84	1.00610	57.4491	0.4	17

Figure S3. MALDI-ToF spectrogram of star-shaped oxypropylene glycol.
